# Supplementary material for: The Systems Biology Research Tool: evolvable open-source software
Source: BMC Syst Biol. 2008 Jun 29;2:55. doi: 10.1186/1752-0509-2-55 (PMC2446383; doi:10.1186/1752-0509-2-55)
Supplement: Additional file 1 — SBRT Archive. An archive of the current version of the Systems Biology Research Tool. [file 1752-0509-2-55-S1.zip › sbrt-1.4.0/doc/developers_guide/api/sbrt/kernel/math/algebra/package-summary.html]

sbrt.kernel.math.algebra


|  |  |  |
| --- | --- | --- |
| **Overview** \| **Package** \| Class \| **Use** \| **Tree** \| **Deprecated** \| **Index** \| **Help** | |  |
| **PREV PACKAGE**   **NEXT PACKAGE** | **FRAMES**    **NO FRAMES**     **All Classes** |  |


---

## Package sbrt.kernel.math.algebra

Provides classes and interfaces for creating objects relevant to algebra.

**See:**
  
          **Description**

| **Interface Summary** | |
| --- | --- |
| **DoubleMatrix<R,C>** | This interface is used to represent matrices whose elements are strictly double precision numbers. |
| **DoubleMatrixBuilder<R,C>** | This interface is used to represent mutable matrices whose elements are strictly double precision numbers. |
| **LinearSystemSolution<V>** | This interface is used to represent a general solution to a system of linear equations. |
| **LinearSystemSolver<V>** | This interface is used to represent classes that solve systems of linear equations. |
| **Matrix<R,C,E>** | This interface is used to represent matrices. |
| **MatrixBuilder<R,C,E>** | This interface is used to represent mutable matrices. |
| **SmdMatrix<R,C>** | This interface is used to represent sparse mutable matrices containing strictly double precision numbers as elements. |
| **SparseDoubleMatrix<R,C>** | This interface is used to represent sparse matrices whose elements are strictly double precision numbers. |

| **Class Summary** | |
| --- | --- |
| **AbstractMdMatrix<R,C>** | This abstract class is a skeleton implementation of `DoubleMatrixBuilder`. |
| **AbstractSmdMatrix<R,C>** | This abstract class is a skeleton implementation of `SmdMatrix`. |
| **DoubleMatrixWrap<R,C>** | This class is used to wrap mutable matrices as an immutable `DoubleMatrix`. |
| **HashLinearSystemSolution<V>** | This class is a concrete implemenation of `LinearSystemSolution`. |
| **HashMatrix<R,C>** | This class is a `java.util.LinkedHashMap` based implementation of `SmdMatrix`. |
| **IndexPair<R,C>** | This class is used to represent pairs of matrix indices. |
| **MatrixElement<R,C,E>** | This class is used to represent matrix elements. |
| **MatrixMultiplier** | This class is used to multiply matrices. |
| **MatrixTransposer** | This class is used to transpose matrices. |
| **MatrixWrap<R,C,E>** | This class is used to wrap mutable matrices as an immutable matrix. |
| **RowOrTreeMatrix<R extends Comparable<? super R>,C extends Comparable<? super C>>** | This class is a `java.util.TreeMap` based implementation of `SmdMatrix`. |
| **SparseDoubleMatrixWrap<R,C>** | This class is used to wrap mutable matrices as an immutable `SparseDoubleMatrix`. |
| **TreeMatrix<R extends Comparable<? super R>,C extends Comparable<? super C>>** | This class is a `java.util.TreeMap` based implementation of `SmdMatrix`. |

| **Exception Summary** | |
| --- | --- |
| **IllegalFreeVariableException** | This class is used to indicate the presence of an illegal free variable. |

## Package sbrt.kernel.math.algebra Description

Provides classes and interfaces for creating objects relevant to algebra.

---


|  |  |  |
| --- | --- | --- |
| **Overview** \| **Package** \| Class \| **Use** \| **Tree** \| **Deprecated** \| **Index** \| **Help** | |  |
| **PREV PACKAGE**   **NEXT PACKAGE** | **FRAMES**    **NO FRAMES**     **All Classes** |  |


---
